# Constructing cancer patient-specific and group-specific gene networks with multi-omics data

**DOI:** 10.1186/s12920-020-00736-7

**Published:** 2020-08-27

**Authors:** Wook Lee, De-Shuang Huang, Kyungsook Han

**Affiliations:** 1 Department of Computer Engineering, Inha University, Incheon, 22212 South Korea; 2 Institute of Machine Learning and Systems Biology, School of Electronics and Information Engineering, Tongji University, Shanghai, 201804 China

**Keywords:** Individual-specific gene network, Group-specific gene network, Cancer, Multi-omics data

## Abstract

**Background:**

Cancer is a complex and heterogeneous disease with many possible genetic and environmental causes. The same treatment for patients of the same cancer type often results in different outcomes in terms of efficacy and side effects of the treatment. Thus, the molecular characterization of individual cancer patients is increasingly important to find an effective treatment. Recently a few methods have been developed to construct cancer sample-specific gene networks based on the difference in the mRNA expression levels between the cancer sample and reference samples.

**Methods:**

We constructed a patient-specific network with multi-omics data based on the difference between a reference network and a perturbed reference network by the patient. A network specific to a group of patients was obtained using the average change in correlation coefficients and node degree of patient-specific networks of the group.

**Results:**

In this paper, we present a new method for constructing cancer patient-specific and group-specific gene networks with multi-omics data. The main differences of our method from previous ones are as follows: (1) networks are constructed with multi-omics (mRNA expression, copy number variation, DNA methylation and microRNA expression) data rather than with mRNA expression data alone, (2) background networks are constructed with both normal samples and cancer samples of the specified type to extract cancer-specific gene correlations, and (3) both patient individual-specific networks and patient group-specific networks can be constructed. The results of evaluating our method with several types of cancer show that it constructs more informative and accurate gene networks than previous methods.

**Conclusions:**

The results of evaluating our method with extensive data of seven cancer types show that the difference of gene correlations between the reference samples and a patient sample is a more predictive feature than mRNA expression levels and that gene networks constructed with multi-omics data show a better performance than those with single omics data in predicting cancer for most cancer types. Our approach will be useful for finding genes and gene pairs to tailor treatments to individual characteristics.

## Background

For the past years, we have witnessed the rapid development of targeted cancer therapy. Targeted therapies for cancer work by targeting specific genes, proteins or tissues that contribute to cancer growth and survival. Many targeted therapies are effective only for patients with specific genetic alterations (known as driver mutations) that help cancer cells form and grow [1, 2]. Thus, identifying genetic mutations specific to individual patients is of utmost importance to determine targeted therapies that can effectively cure cancer patients while minimizing side effects [3].

Motivated by a massive amount of data generated by high-throughput technologies, several cancer studies used gene networks to explore gene expression characteristics [4–8]. However, constructing a patient-specific gene network with a single sample obtained from a patient is difficult because a gene network requires many samples to compute gene-gene relations.

Recently a few methods have been proposed to construct cancer sample-specific gene networks based on the difference in the mRNA expression levels between the cancer sample and reference samples. For example, Liu et al. [9] proposed a method to construct a sample-specific network by computing the difference between a reference network from multiple reference samples and a network perturbed by a new sample. However, a slight change to the reference samples can result in a significantly different sample-specific network for the same sample due to the small number of reference samples. Furthermore, their sample-specific networks cannot reflect post-translational modification and epigenetics because the networks are built using mRNA expression data only.

This paper presents a new method for constructing cancer patient-specific and group-specific gene networks with multi-omics data using a sample-specific network and network propagation method. Network propagation strategies are widely used in recent cancer-related research. Li et al. [10] presented a synergy prediction algorithm using network propagation and predicted the drug synergy in various cancers. Zhang et al. [11] introduced a propagation algorithm, which learns the mutated subnetworks underlying tumor subtypes using a supervised approach and classified tumors to known subtypes on breast and glioblastoma tumors. Peng et al. [12] identified bladder cancer-related genes by propagating information from seed genes to candidate genes. The primary focus of our method is to construct a gene correlation network specific to cancer with multi-omics data. Thus, it is different from a typical gene co-expression network that represents co-expression relations between genes from mRNA expression data. Our gene network is not a gene regulatory network because our network does not show regulatory relations between genes.

The main differences of our method from previous ones are as follows: (1) networks are constructed with multi-omics (mRNA expression, copy number variation, DNA methylation and microRNA expression) data rather than with mRNA expression data alone, (2) background networks are constructed with both normal samples and cancer samples of the specified type to extract cancer-specific gene correlations, and (3) both patient individual-specific networks and patient group-specific networks can be constructed. As shown later in this paper, the results of evaluating our method with several types of cancer show that it constructs more informative and accurate target-specific networks than previous methods.

## Methods

**Fig. 1:**
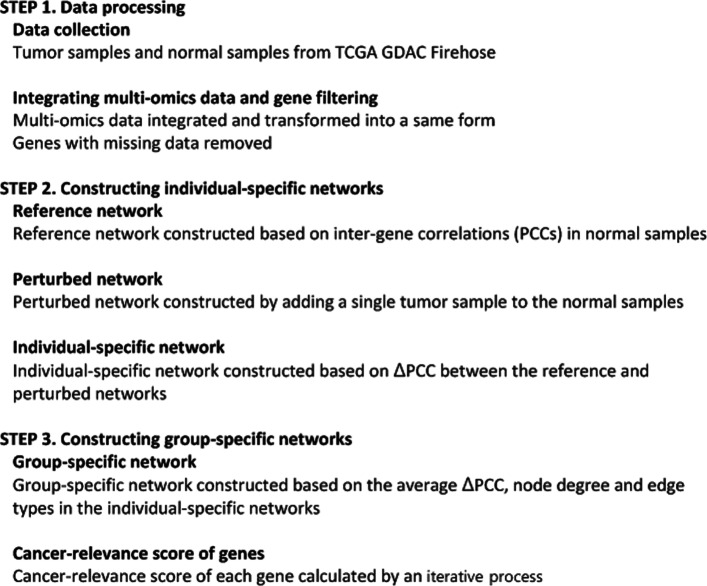
Overview of constructing an individual-specific network and a group-specific network with multi-omics data

At the top level, our method consists of the following steps: (1) data processing, (2) constructing individual-specific gene networks, and (3) constructing a group-specific gene networks. A high-level description of the method is given in Fig. 1.

### Data collection and preprocessing

From the Broad Institute TCGA GDAC Firehose [13], we obtained multi-omics data of cancer samples of seven types: breast invasive carcinoma (BRCA), colon adenocarcinoma (COAD), head and neck squamous cell carcinoma (HNSC), pan-kidney cohort (KIPAN), liver hepatocellular carcinoma (LIHC), lung adenocarcinoma (LUAD) and lung squamous cell carcinoma (LUSC).

The multi-omics data used in this study include mRNA expression (mRNAseq), copy number variation (CNV), DNA methylation and mature miRNA expression (miRseq) data. The mRNAseq data were processed using quartile normalized RSEM [14] and then log2-transformed. The segmented CNV data were converted to gene-level data using the Ensembl API [15] and the CNTools package [16] of Bioconductor. The methylation data were filtered to select the probe with the mean signal values for each gene. The miRseq data were processed by RPM and log2-transformed. mRNAs and miRNAs that were not expressed in more than 10% of the total samples were excluded in further analysis. Missing expression values of mRNAs and miRNAs were replaced by the smallest positive normalized floating-point number (realmin) of MATLAB. The number of samples and genes used in this study are available in Additional file 1.

### Individual-specific gene network

**Fig. 2:**
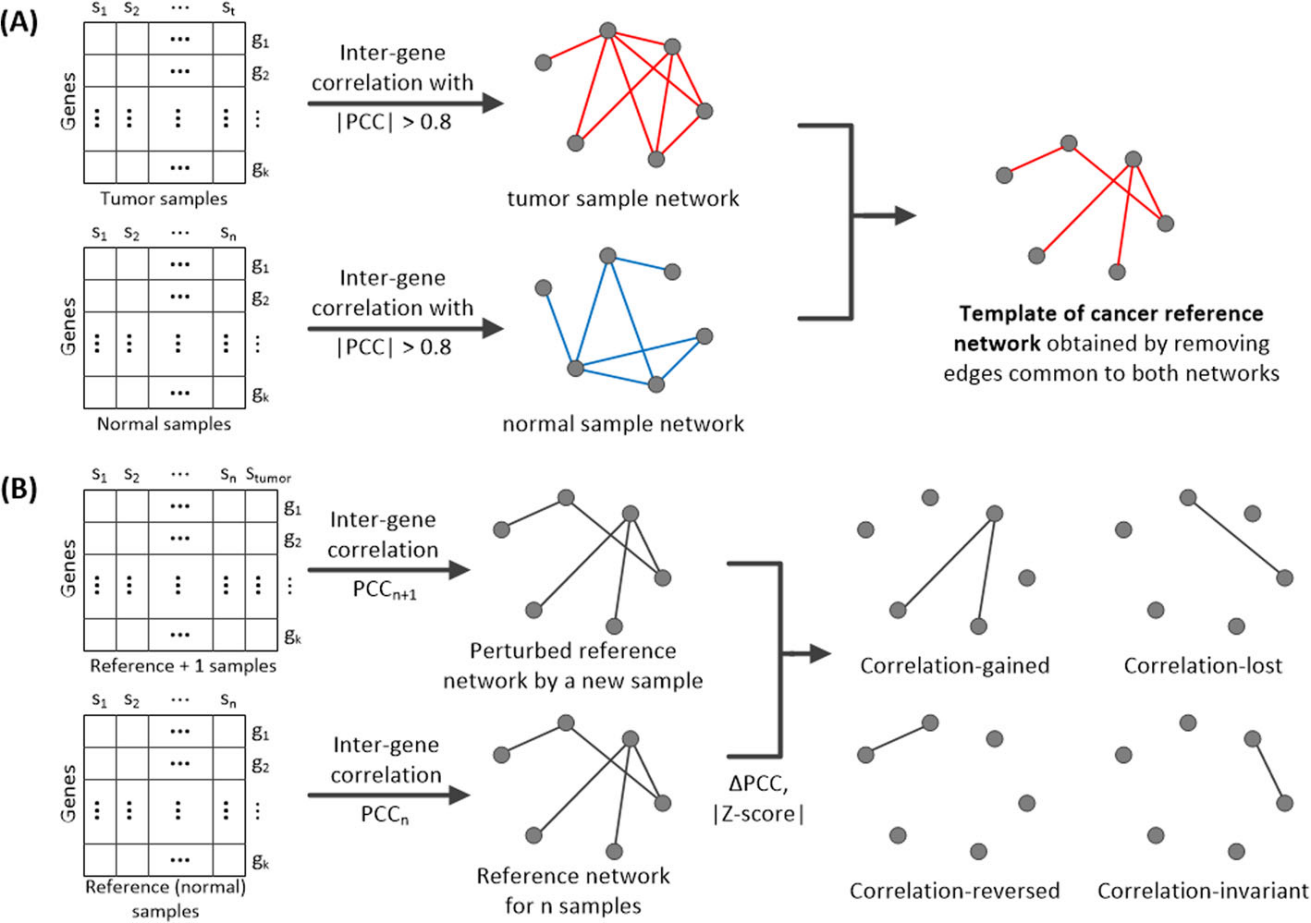
Process of constructing a patient-specific gene network. **a** template of the cancer reference network obtained by removing edges common to both networks. **b** patient-specific gene network and four types of edges in the network. Edges with a $\left |, \text {Z-score} \right |, $ < 1 represent correlation-invariant gene pairs, and edges with $\left |, \text {Z-score} \right |, \geq $ 1 and different signs of *PCC*_*n*_ and *PCC*_*n*+1_ represent correlation-reversed gene pairs. Edges with $\left | \text {Z-score} \right | \geq $ 1 and *ΔPCC*>0 are correlated-gained gene pairs, and those with $\left | \text {Z-score} \right | \geq $ 1 and *ΔPCC*<0 are correlation-lost gene pairs

In each group of tumor samples and normal samples, we first computed gene-gene relations by the Pearson correlation coefficient (PCC), selected highly correlated gene pairs (i.e., those with $\left | PCC \right | >$0.8), and constructed two sample networks, one for each group. From the tumor sample network, we removed edges common to both tumor and normal sample networks and obtained a template reference network for cancer (Fig. 2a). The template reference network consists of highly-correlated gene pairs that are specific to cancer.

With *n* reference samples, which may be different from tumor samples used in the template network, we computed PCC for every pair of genes in the template reference network and constructed a reference network for the reference samples. For a patient of interest, we constructed a network, which is a perturbed network by adding a single sample of the patient to the *n* reference samples. A patient-specific network was obtained by subtracting the reference network from the perturbed network.

1 $$ \Delta PCC = \left| PCC_{n+1} \right| - \left| PCC_{n} \right|   $$

We computed the difference in the absolute value of PCC between the perturbed reference network and reference network by Eq. 1. We also carried out a Z-test of *PCC*_*n*+1_−*PCC*_*n*_ by Eq. 2. For a large *n*, we can approximate the mean (*μ*) and standard deviation (*σ*) of *PCC*_*n*+1_−*PCC*_*n*_ as 0 and $(1 - PCC_{n}^{2})/(n - 1)$, respectively [9].

2 $$ \begin{aligned} PCC_{change} &= PCC_{n+1}-PCC_{n} \\ Z-score &= \frac{PCC_{change} - \mu (PCC_{change})}{\sigma (PCC_{change})} = \frac{PCC_{change}}{\frac{1 - PCC_{n}^{2}}{n - 1}}  \end{aligned}  $$

The edges of the patient-specific network were classified into four types [9]: (1) correlation-gained edges for gene pairs whose PCCs are increased from the reference network to the patient-specific network, (2) correlation-lost edges for gene pairs whose PCCs are decreased from the reference network to the patient-specific network, (3) correlation-reversed edges for gene pairs whose signs of PCCs are changed from positive to negative or negative to positive, and (4) correlation-invariant edges for gene pairs with little change in PCCs between the reference and patient-specific networks (i.e., those with $\left | \text {Z-score} \right | < 1$) (Fig. 2b).

The edges were classified in the following way. We first selected gene pairs with $\left | \text {Z-score} \right | < 1$ as correlation-invariant type, and then selected gene pairs which have different signs of PCCs between the reference network and the patient-specific network as correlation-reversed type. The remaining gene pairs were classified into either correlation-gained or correlation-lost type depending on whether their PCCs are increased (correlation-gained) or decreased (correlation-lost) from the reference network to the patient-specific network. Thus, $\left | \text {Z-score} \right | \geq 1$ in both correlation-gained and correlation-list gene pairs.

### Group-specific gene network

**Fig. 3:**
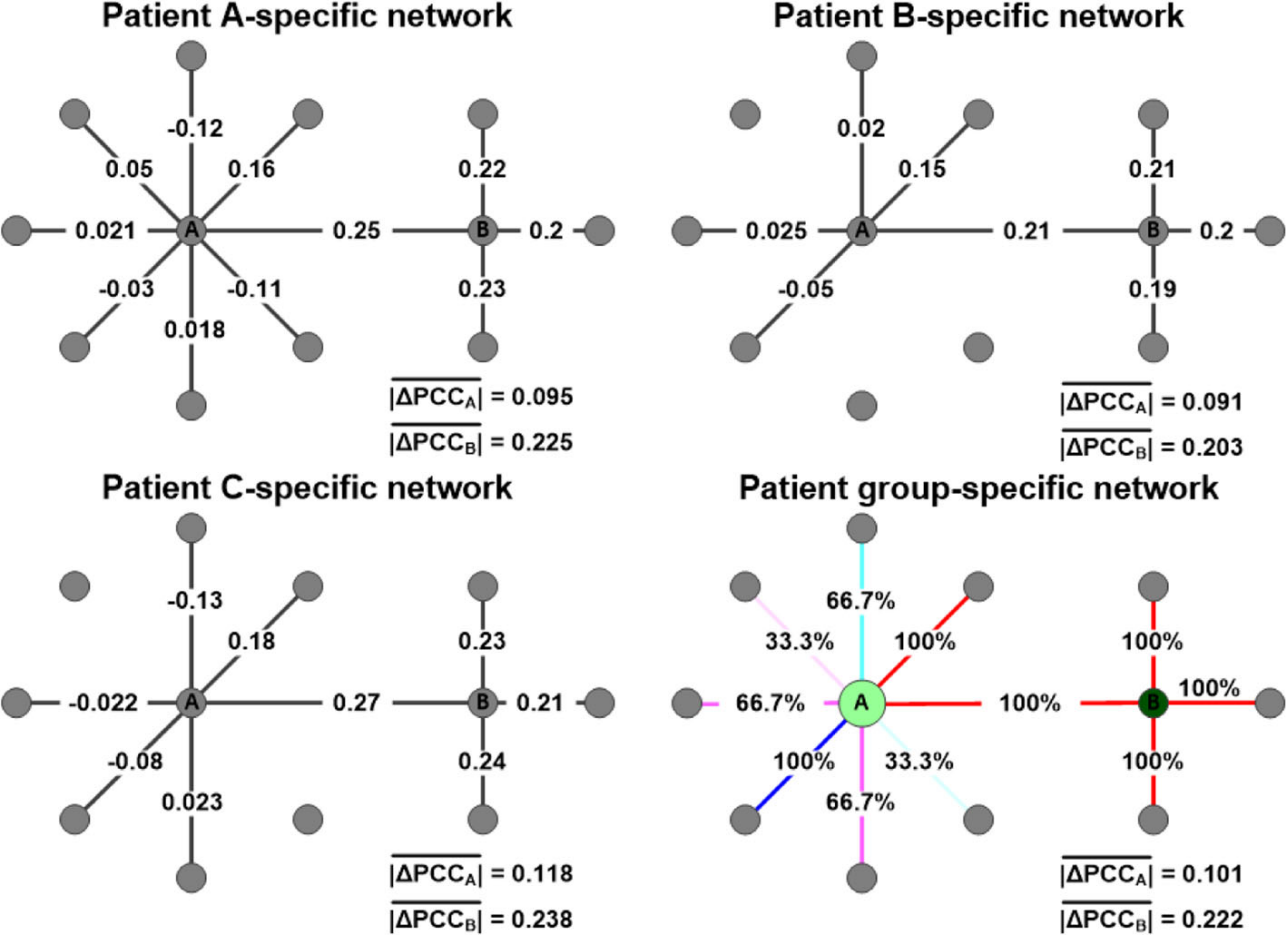
Process of constructing a group-specific gene network from multiple patient-specific gene networks. In the group-specific gene network, the node size and node color are proportional to the average degree and average $\left | \Delta PCC \right |$ of the node, respectively. If the dominant type for a particular edge is ‘correlation-gained’ (positive *Δ*PCC) in the patient-specific networks, the edge is represented in red in the group-specific network. If the dominant type for a particular edge is ‘correlation-lost’ (negative *Δ*PCC) in the patient-specific networks, the edge is represented in blue in the group-specific network

A group-specific gene network is useful when analyzing a large number of patient-specific gene networks. After constructing patient-specific gene networks, we obtained a gene network specific to a group of patients based on the average *Δ*PCC and node degree of the patient-specific networks (Fig. 3). If the dominant type for a particular edge is ‘correlation-gained’ (positive *Δ*PCC) in the patient-specific networks, the edge is represented in red in the group-specific network. In contrast, if the dominant type for a particular edge is ‘correlation-lost’ (negative *Δ*PCC) in the patient-specific networks, the edge is represented in blue in the group-specific network. In the group-specific network, only the dominant type is shown for each edge. If non-dominant types are shown in addition to the dominant type for each edge, the network becomes cluttered and unreadable. The node size of a group-specific gene network is proportional to the average degree of the node.

### Integration of multi-omics data

To integrate multi-omics data, we first computed inter-gene correlations by PCC with four different types of single omics data (mRNA expression, CNV, DNA methylation and miRNA expression) separately, and selected significant inter-gene correlations only. In mRNA expression, CNV and DNA methylation data, we select the top 1% $\left | PCC \right |$ with *p*-value <0.01. In miRNA expression data, we selected the top 5% $\left | PCC \right |$ with *p*-value <0.01 due to a smaller number of miRNAs in the data. The inter-gene correlations selected in each single omics data are represented in four correlation matrices (*M*_*expr*_, *M*_*CNV*_, *M*_*methyl*_ and *M*_*miRNA*_) and normalized.

Using the protein-protein interactions (PPIs) of the STRING database [17], we constructed separate weighted networks from each omics data by Eq. 3. In Eq. 3, *W*_*expr*_, *W*_*CNV*_ and *W*_*methyl*_ denote the weighted networks, and *PPI*_*expr*_, *PPI*_*CNV*_ and *PPI*_*methyl*_ are subnetworks of a PPI network consisting of genes present in each omics data. Since the PPI network does not contain information on miRNA, a weighted network for miRNA was not constructed.

3 $$ \begin{aligned} W_{expr} &= 1 - \left(1 - M_{expr}\right) \times \left(1 - PPI_{expr}\right) \\[-3pt] W_{CNV} &= 1 - \left(1 - M_{CNV}\right) \times \left(1 - PPI_{CNV}\right) \\[-3pt] W_{methyl} &= 1 - \left(1 - M_{methyl}\right) \times \left(1 - PPI_{methyl}\right)  \end{aligned}  $$

We then integrated the multi-omics data by linear regression using Eq. 4 [12]. In Eq. 4, *Y*_*i*_, $X^{CNV}_{i}$, $X^{methyl}_{i}$ and $X^{miRNA}_{ij}$ denote gene *i*’s expression level, CNV level, methylation level, and miRNA regulator expression level, respectively. $\beta ^{CNV}_{i}$ and $\beta ^{methyl}_{i}$ denote the regression coefficients of gene *i*’s expression level on CNV and methylation, respectively. $\beta ^{miRNA}_{ij}$ is the regression coefficient of gene *i*’s expression level on its miRNA regulator *j*’s expression level.

4 $$ Y_{i} \,=\, \beta^{CNV}_{i} X^{CNV}_{i} \,+\, \beta^{methyl}_{i} X^{methyl}_{i} \,+\, \sum\limits_{j=1}^{n} {\beta^{miRNA}_{ij} X^{miRNA}_{ij}} + \epsilon   $$

From the regression coefficients and the weighted networks, a weight matrix *W* was derived and normalized into $\overline {W}$ (Eqs. 5 and 6). The weight matrix *W* is symmetric, so *W*_*ij*_=*W*_*ji*_. *W*_11_, *W*_22_, *W*_33_ and *W*_44_, represent *W*_*expr*_, *W*_*CNV*_, *W*_*methyl*_ and *M*_*miRNA*_, respectively. The submatrices *W*_21_ and *W*_31_ contain regression coefficients $\beta ^{CNV}_{i}$ and $\beta ^{methyl}_{i}$ for every gene *i*, respectively. *W*_41_ represents $\beta ^{miRNA}_{ij}$. The submatrices *W*_32_, *W*_42_ and *W*_43_ are empty.

5 $$ W = \left[\begin{array}{llll} W_{11} & W_{12} & W_{13} & W_{14} \\ W_{21} & W_{22} & W_{23} & W_{24} \\ W_{31} & W_{32} & W_{33} & W_{34} \\ W_{41} & W_{42} & W_{43} & W_{44} \end{array}\right]   $$

6 $$ \overline W(i, j) = W(i, j)/\sqrt{\sum\limits_{k=1}^{m} W(i, k) \times \sum\limits_{k=1}^{m} W(k, j)}   $$

In network propagation, seed genes have greater impact than non-seed genes on their neighbors. Thus, only the genes with a high average *Δ*PCC were selected as seed genes for a group-specific network, and their miRNAs regulators extracted from miRTarBase [18] were used as seed miRNAs. We calculated the cancer-relevance *S*^*t*^ of each gene to reflect the effect of the seed genes and miRNAs on neighbors. The initial score *D* was calculated by Eq. 7 and updated iteratively by Eq. 8 [12]. In this iterative process, the influence of the seed is propagated to the neighbors until a mean squared error of *S*^*t*^ and *S*^*t*−1^ ≤1×10^−5^.

7 $$ D_{v} = \left\{\begin{array}{ll} \frac{n_{v}}{N_{v}} & \text{if} {v} \text{is a non-seed} \& N_{v} \geq \alpha \\ e^{N_{v} - \alpha} \times \frac{n_{v}}{N_{v}} & \text{if} {v} \text{is a non-seed} \& N_{v} < \alpha \\ 1 & \text{if} {v} \text{is a seed} \end{array}\right.   $$

8 $$ S^{t} = \lambda \times S^{t-1} \times \overline{W} + (1 - \lambda) \times D \text{, where} S^{1} = D   $$

where *N*_*v*_ is the number of neighbors of node *v*, and *n*_*v*_ is the number of seeds in the neighbors. The parameter *α*, which is a threshold for *N*_*v*_, was set to 50 and *λ* was set to 0.2 [12]. Genes with the top 10% *S*^*t*^ were used in finding cancer-related genes and in classifying tumor samples and normal samples.

## Results

### Patient-specific and group-specific gene networks

**Fig. 4:**
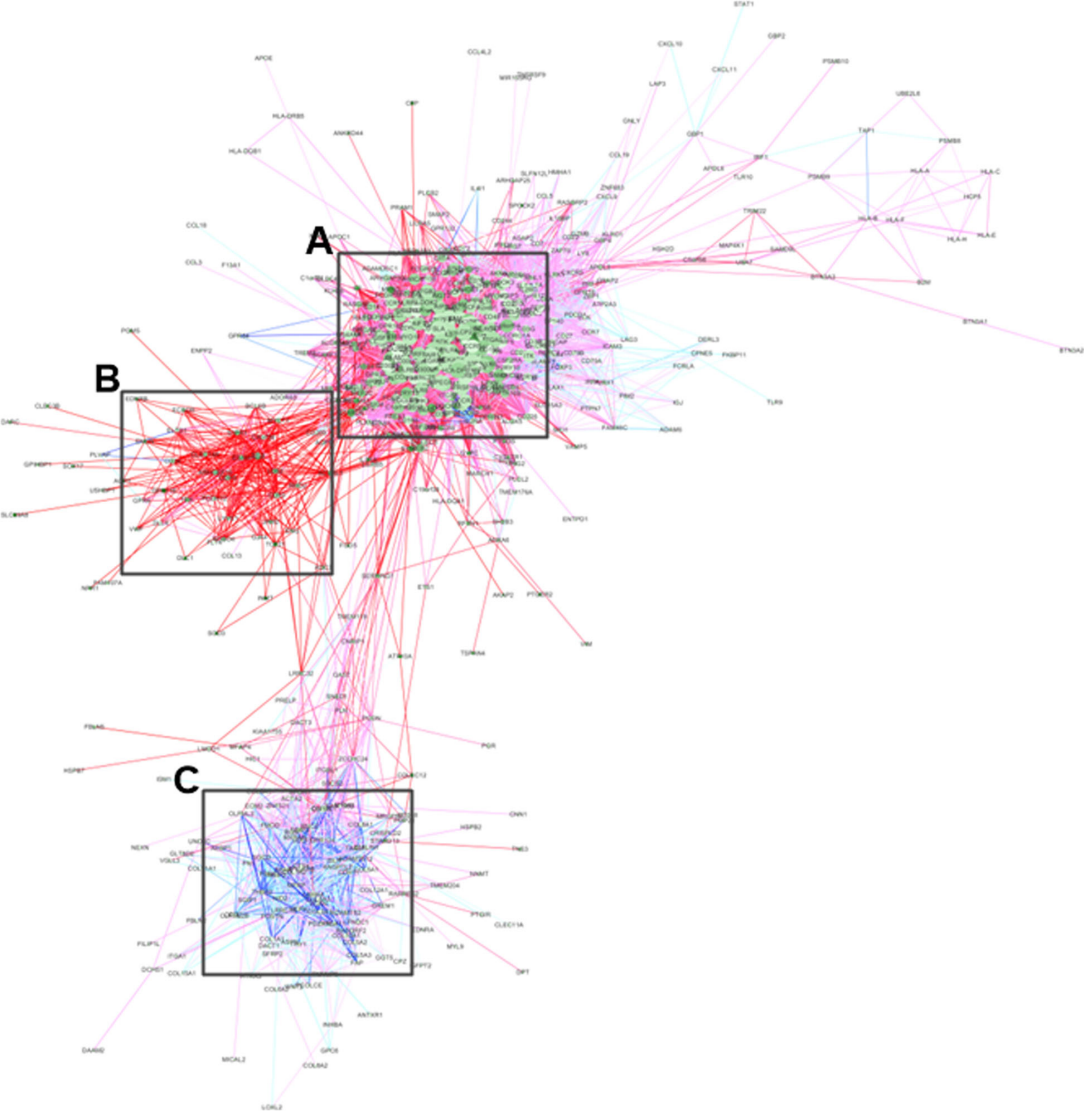
Group-specific gene network for 300 lung squamous cell carcinoma (LUSC) patients. **a** subnetwork of multiple hub genes (large green nodes). **b** subnetwork of correlation-gained edges (dark red edges). **c** subnetwork with many correlation-lost edges (dark blue edges)

In this study, we constructed 2,400 patient-specific gene networks for seven cancer types (Additional file 1). For each cancer type, we also constructed group-specific gene networks. As an example, Fig. 4 shows a group-specific gene network derived from 300 lung squamous cell carcinoma (LUSC) patients.

There are three distinct subnetworks in the network for the LUSC group. The subnetwork enclosed in box A of Fig. 4 contains many hub genes (large green nodes). The subnetwork in box B consists of correlation-gained edges (dark red edges), whereas the subnetwork in box C contains many correlation-lost edges (dark blue edges).

### Comparison of multi-omics data and single-omics data

We performed leave-one-out cross validation (LOOCV) to evaluate cancer-relevance score *S*^*t*^ of a gene and the contribution of multi-omics data to finding cancer-related genes. For comparison, the cancer-relevance scores were computed with multi-omics data and single omics data separately. Each seed gene was regarded as a non-seed and a new cancer-relevance score was calculated for the gene. Seed genes and non-seed genes were considered as positive and negative, respectively. Seed genes included in the top *n* genes were considered as true positives, and seed genes not included in the top *n* genes were considered as false negatives. Similarly, non-seed genes included in the top *n* genes and non-seed genes not included in the top *n* genes were considered as false positives and true negatives, respectively.

**Fig. 5:**
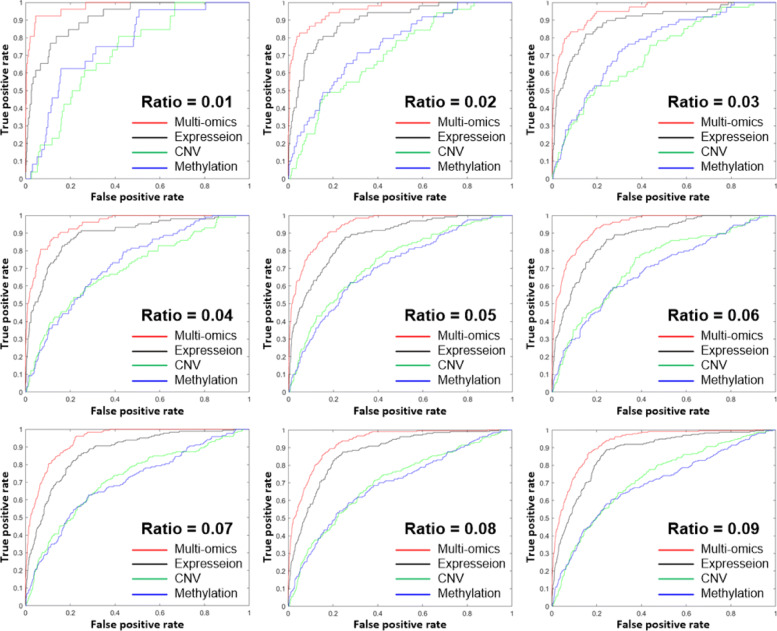
ROC curves of the leave-one-out cross validation of the cancer relevance score *S*^*t*^ of genes with different ratios of seed genes. 400 breast cancer samples were used in the leave-one-out cross validation. The performance of multi-omics data is always better than that of single omics data

We carried out LOOCV with different ratios of seed genes to non-seed genes. Figure 5 shows the receiver operating characteristic (ROC) curve and the area under the curve (AUC) of LOOCV of the cancer relevance of genes on data of 400 breast cancer samples with various seed ratios ranging from 0.01 to 0.09 (Enlarged plots of Fig. 5 are available in Additional file 2). For comparative purposes, we also computed the cancer relevance of genes with single omics data. As shown in Fig. 5, multi-omics data consistently exhibited better performance than single omics data with any seed ratio between 0.01 to 0.09. For later analysis, the seed ratio was set to 0.05 by default. The average *Δ*PCC and class label of each gene are available in Additional file 3.

Indeed, the superiority of multi-omics data over single omics data in determining the cancer relevance score of genes was observed in all seven types of cancer (Additional file 4). In seven types of cancer, the cancer relevance score of genes computed with multi-omics data exhibited a good performance (AUC = 0.896 ∼0.942). The cancer relevance score of genes computed with mRNA expression data showed the second best performance (AUC = 0.761 ∼0.878). In particular, the cancer relevance score computed with mRNA expression data showed a very similar performance to that with multi-omics in breast cancer (BRCA). The performance of the cancer relevance score computed with CNV (AUC = 0.591 ∼0.786) and DNA methylation data (AUC = 0.581 ∼0.817) alone was lower than that with mRNA expression data (AUC = 0.761 ∼0.878).

### Evaluation of gene correlations and background networks

Many network-based approaches to cancer research have focused on finding genes that show differential expressions between tumor samples and normal samples. Gene-gene correlations (i.e., inter-gene correlations) may be more helpful than individual genes because inter-gene correlations depend on the expression of neighbor genes in a gene regulatory network. To compare the effect of using individual genes to that of inter-gene correlations (i.e., *Δ*PCC), we constructed a support vector machine (SVM) model for classifying cancer samples and normal samples. The SVM model was implemented using C-SVC and RBF kernel, and the parameter values of the model were determined by the grid search algorithm. mRNA expression levels and *Δ*PCCs were used as features of the SVM models. For rigorous validation, the test data used in testing the models were not used in training them (Additional file 1).

**Fig. 6:**
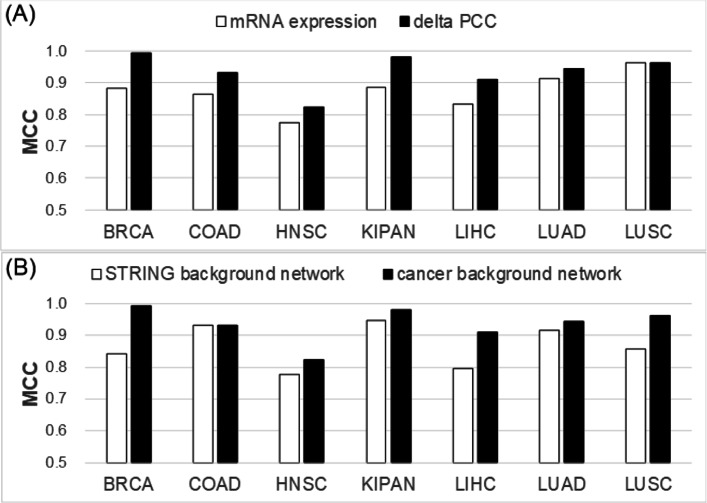
Results of evaluating features and background networks by a validation set. **a** Comparison of mRNA expressions of genes and *Δ*PCC of gene pairs. **b** Comparison of the cancer background network with the background network from PPI data

As shown in Fig. 6a, *Δ*PCC showed a better performance than mRNA expression levels for six cancer types except LUSC. The classification model with *Δ*PCC showed MCC above 0.9 in six cancer types except HNSC.

We also examined the effect of different background networks on individual-specific networks. In the work by Liu et al. [9], PPI data with high confidence scores in the STRING database were used to construct a background network. However, the PPI data of STRING does not reflect cancer type-specific characteristics. Figure 6b shows the performance of the classification model with two different background networks: background network from PPI data of STRING (the approach by Liu et al.) and cancer background network (our approach). *Δ*PCC was used as a feature of the classification model. Except for COAD, the performance of the classification model with the cancer background network was better than the model with the STRING reference background network. In particular, the classification model showed a significant difference for breast cancer (BRCA) (MCC of 0.992 vs. MCC of 0.841). Detailed results of the classification model are available in Additional file 5.

## Discussion

In the analysis of finding cancer-related genes and gene pairs, we focused on a subnetwork of genes with a *Δ*PCC. Table 1 shows the top 10 genes with a high average *Δ*PCC in each group-specific network of seven cancer types. In breast invasive carcinoma (BRCA), FAM171A1 showed the highest average *Δ*PCC in the group-specific network. FAM171A1 is known as a potential biomarker in triple-negative breast cancer [19]. FOXC1 is involved in tumor development and metastasis and associated with poor prognosis in basal-like breast cancer [20]. IL-33 is overexpressed in various cancers and the serum concentration of IL-33 is a valuable indicator of poor prognosis in breast cancer. [21]. MAMDC2 is significantly correlated with disease-free survival of breast cancer patients [22]. MTERFD1 is closely related to breast cancer recurrence [23] and HOXA7 plays a critical role in regulating the proliferation of ER-positive cancer cells [24].

**Table 1:** Top 10 genes with a high average *Δ*PCC in a group-specific network for seven cancer types

**BRCA**	**COAD**	**HNSC**	**KIPAN**	**LIHC**	**LUAD**	**LUSC**
FAM171A1	GFRA2	CYP2J2	TFCP2L1	SLC19A3	CLDN18	NSUN2
FOXC1	SCNN1B	BARX2	KCNQ1	ECM1	ADAMTS8	CCT5
IL33	DDX27	ZNF135	ARL15	CYP2B6	PECAM1	FBXO45
MAMDC2	RNPS1	PPFIA1	KCTD1	FBP1	SFTPA1	GPR116
MTERFD1	UBE2I	PARL	OAZ2	GPAA1	GIMAP6	SLC39A8
HOXA7	MDFIC	FADD	TMEM45B	F9	AKR1C1	FXR1
CTTNBP2	CTNNBL1	CST6	HPCAL1	PAH	MME	INMT
ZNF204P	CDK5RAP1	COBL	TMEM91	AGXT2	ATAD2	VEPH1
JAM3	ESF1	ORAOV1	SEMA5B	HSD17B6	CYP3A5	CRTAM
FREM1	TRMT6	XPO7	EGLN3	RNASE4	CHAF1B	WDR53

In colon adenocarcinoma (COAD), GFRA2 showed the highest average *Δ*PCC in the group-specific network. It is known to be crucial for enteric neuron survival [25]. SCNN1B and DDX27 are significantly related to colorectal cancer [26, 27]. No direct relation of RNPS1 with colorectal cancer is known, but RNPS1 is essential to nonsense-mediated mRNA decay [28] that plays complex functions in cancer [29]. Knockdown of SUMO conjugating enzyme UBE2I (also known UBC9 or E2) inhibits maintenance and self-renewal of colorectal cancer stem cell, while overexpression of UBE2I increases colorectal cancer cell stemness [30].

Among the top 10 genes with a high average *Δ*PCC in lung adenocarcinoma (LUAD), several genes such as CLDN18, ADAMTS8, PECAM1 and SFTPA1 have been known to be associated with LUAD in previous studies [31–33]. No direct relation of NSUN2 and SLC39A8 with lung squamous cell carcinoma (LUSC) has been known so far. However, recent studies [34, 35] reported that NSUN2 is correlated with survival in other types of squamous cell carcinomas. Gao et al. also showed that the epigenetic silencing of SLC39A8 expression by DNA methylation is involved in the acquisition of resistance against cadmium in lung cells [36] and the relation between cadmium and lung cancer has received much attention [37]. Many other genes in Table 1 found in the group-specific networks for head and neck squamous cell carcinoma (HNSC), pan-kidney cohort (KIPAN) and liver hepatocellular carcinoma (LIHC) are also directly or indirectly related to cancer.

**Fig. 7:**
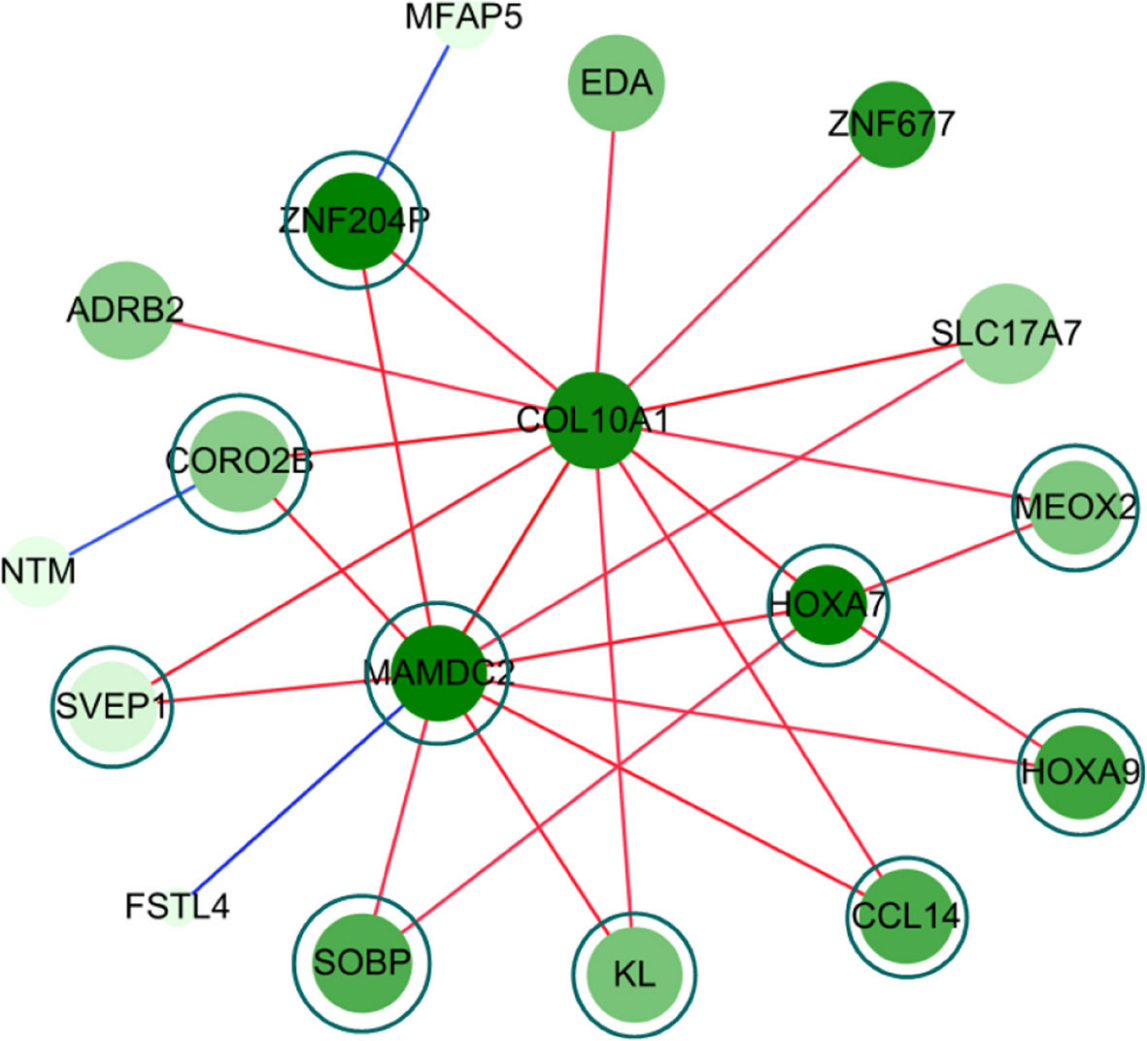
A subnetwork of the group-specific network of BRCA, which contains MAMDC2 and HOXA7. Genes in the most frequent gene pairs shown in Table 2 are enclosed by a circle

In addition to individual genes, we identified gene pairs of the same type (i.e., either correlation-gained or correlation-lost in most patient-specific networks of the same type). Table 2 shows the most frequent gene pairs in 400 breast cancer samples. The most frequent gene pairs in other types of cancer are listed in Additional file 6. It is interesting to note that all the gene pairs shown in Table 2 include at least one gene in the gene pair MAMDC2-HOXA7 and that they are correlation-gained edges in the group-specific network for breast cancer. Figure 7 shows a subnetwork containing MAMDC2 and HOXA7 in the group-specific network of breast cancer. The subnetwork was obtained by selecting the edges for which the proportion of the same edge type (i.e., correlation-gained or lost) is above 90% in the total individual-specific networks of breast cancer patients. It is interesting to note that all the gene pairs in Table 2 are included in the subnetwork.

**Table 2:** The most frequent gene pairs in 400 breast cancer samples. All the gene pairs are of a correlation-gained type. The genes of Table 1 are shown in bold. The proportion represents the ratio of the gene pairs of the same type (i.e., correlation-gained or lost) to the total number of patient-specific networks

Gene pair	#gene pairs		Proportion of the gene pairs
			in total cancer samples
**MAMDC2**-**HOXA7**	380		95.0%
**MAMDC2**-CCL14	379		94.8%
**MAMDC2**-**ZNF204P**	377		94.3%
**MAMDC2**-KL	376		94.0%
**MAMDC2**-SVEP1	376		94.0%
**MAMDC2**-CORO2B	375		93.8%
**HOXA7**-MEOX2	372		93.0%
**HOXA7**-HOXA9	366		91.5%
**MAMDC2**-SOBP	366		91.5%
**MAMDC2**-HOXA9	365		91.3%

To date, the actual role of the MAMDC2 gene in cancer is not clear, but Meng et al. [22] reported MAMDC2 as one of three genes (MAMDC2, TSHZ2, and CLDN11) that are significantly correlated with disease-free survival of breast cancer patients. MAMDC2 is known as a target of miR-196a in head and neck squamous cell carcinoma [38]. As a member of the family of homeobox genes, HOXA7 is associated with cell proliferation, nerve invasion, distant metastasis and degree of tumor differentiation in several cancers [24, 39–42]. HOXA7 is regulated by several miRNAs, including miR-196 [43–45]. Thus, both MAMDC2 and HOXA7 are related with miR-196, but a clear relation among them is to be uncovered.

## Conclusion

So far, most approaches to constructing individual-specific gene networks have been constructed based on the differential expressions between a small number of reference samples and a sample of interest. However, such networks cannot reflect post-translational modification and epigenetics and are not reliable because a slight change to the reference samples can result in a significantly different sample-specific network for the same sample.

In this paper, we presented a new approach to constructing cancer patient-specific and group-specific networks with multi-omics data. The main differences of our method from previous ones are as follows: (1) gene networks are constructed with multi-omics (mRNA expression, copy number variation, DNA methylation and microRNA expression) data rather than with mRNA expression data alone, (2) background networks can be constructed with cancer samples of the specified type, and (3) both patient individual-specific networks and patient group-specific networks can be constructed. The results of testing our method with several cancer types showed that it constructs more informative and accurate gene networks than existing methods.

Evaluation of our method with extensive data of seven cancer types showed that changes in gene correlations (*Δ*PCC) between the reference samples and a patient sample is a more predictive feature than mRNA expression levels and that gene networks constructed with multi-omics data are more powerful than those with single omics data in predicting cancer for most cancer types. More work is required to validate the genes and gene pairs identified in the cancer patient-specific and group-specific networks. However, the method for constructing networks specific to individual patients or patient groups with multi-omics data should be useful aids in determining effective treatments to individual characteristics.

## Supplementary information

**Additional file 1** Number of samples and genes in 7 types of cancer.

**Additional file 2** ROC curve and AUC of the cancer-relevance score of BRCA by various seed ratios.

**Additional file 3** Average *Δ*PCC and class label of each gene in 7 types of cancer.

**Additional file 4** ROC curve of the cancer-relevance score of each cancer type with the seed ratio of 0.05.

**Additional file 5** Performance of classification of tumor samples and normal samples.

**Additional file 6** Top 10 gene pairs for each cancer type.

## Data Availability

Additional files are available at http://bclab.inha.ac.kr/CancerNetwork.

## Abbreviations

AUC: Area under the curve
BRCA: Breast invasive carcinoma
CNV: Copy number variation
COAD: Colon adenocarcinoma
GDAC: Genome data analysis center
HNSC: Head and neck squamous cell carcinoma
KIPAN: Pan-kidney cohort
LIHC: Liver hepatocellular carcinoma
LOOCV: Leave-one-out cross validation
LUAD: Lung adenocarcinoma
LUSC: Lung squamous cell carcinoma
MCC: Matthews correlation coefficient
PCC: Pearson correlation coefficient
PPI: Protein-protein interactions
ROC: Receiver operating characteristic
SVM: Support vector machine
TCGA: The cancer genome atlas
