# Supplementary figures and images for: Constructing cancer patient-specific and group-specific gene networks with multi-omics data
Source: BMC Med Genomics. 2020 Aug 27;13(Suppl 6):81. doi: 10.1186/s12920-020-00736-7 (PMC7450550; doi:10.1186/s12920-020-00736-7)

**Additional file 2.**

**ROC curve and AUC of cancer-relevance score of BRCA by various seed ratios.**

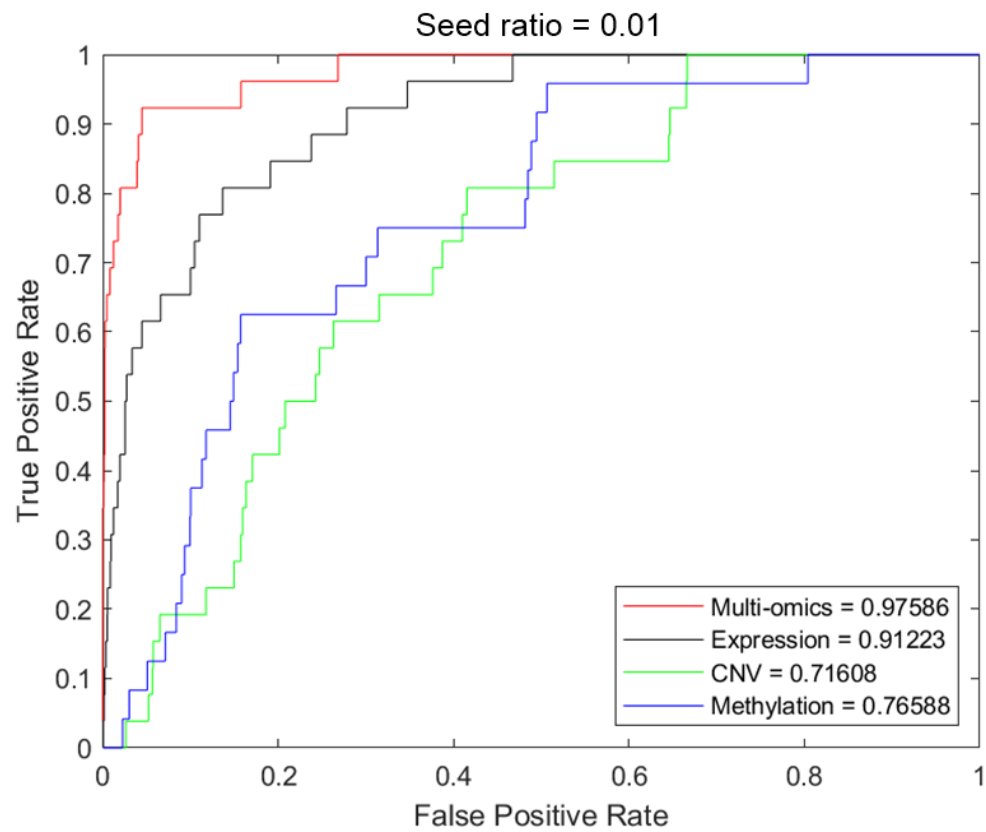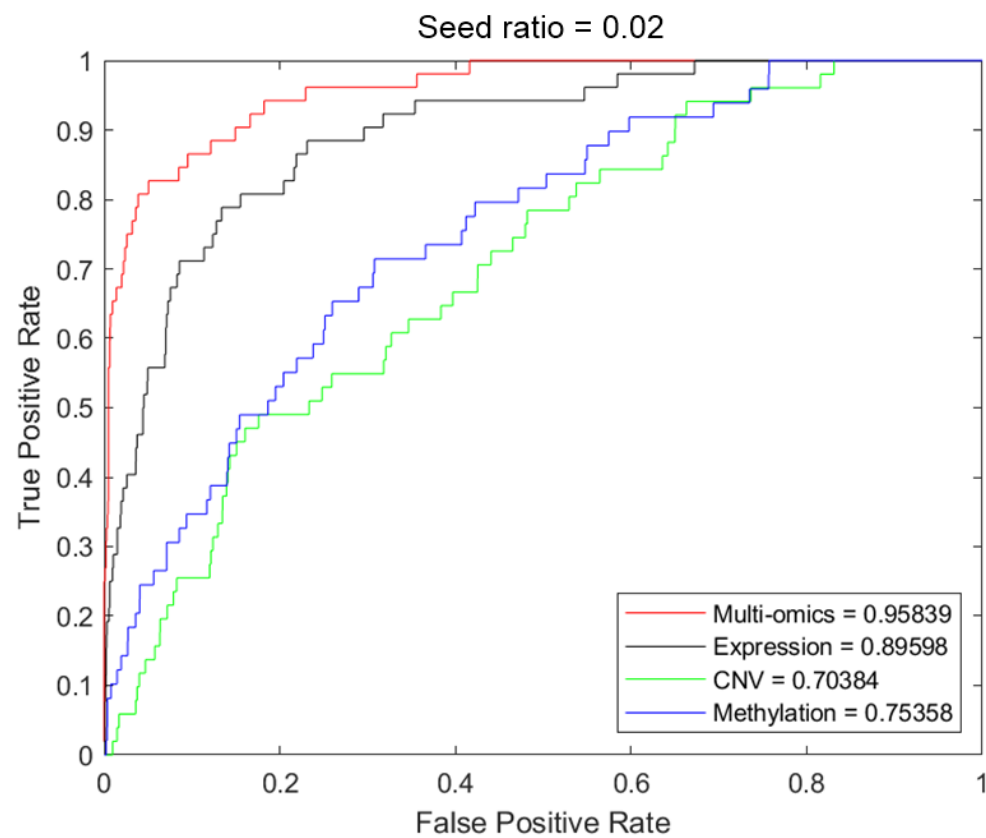

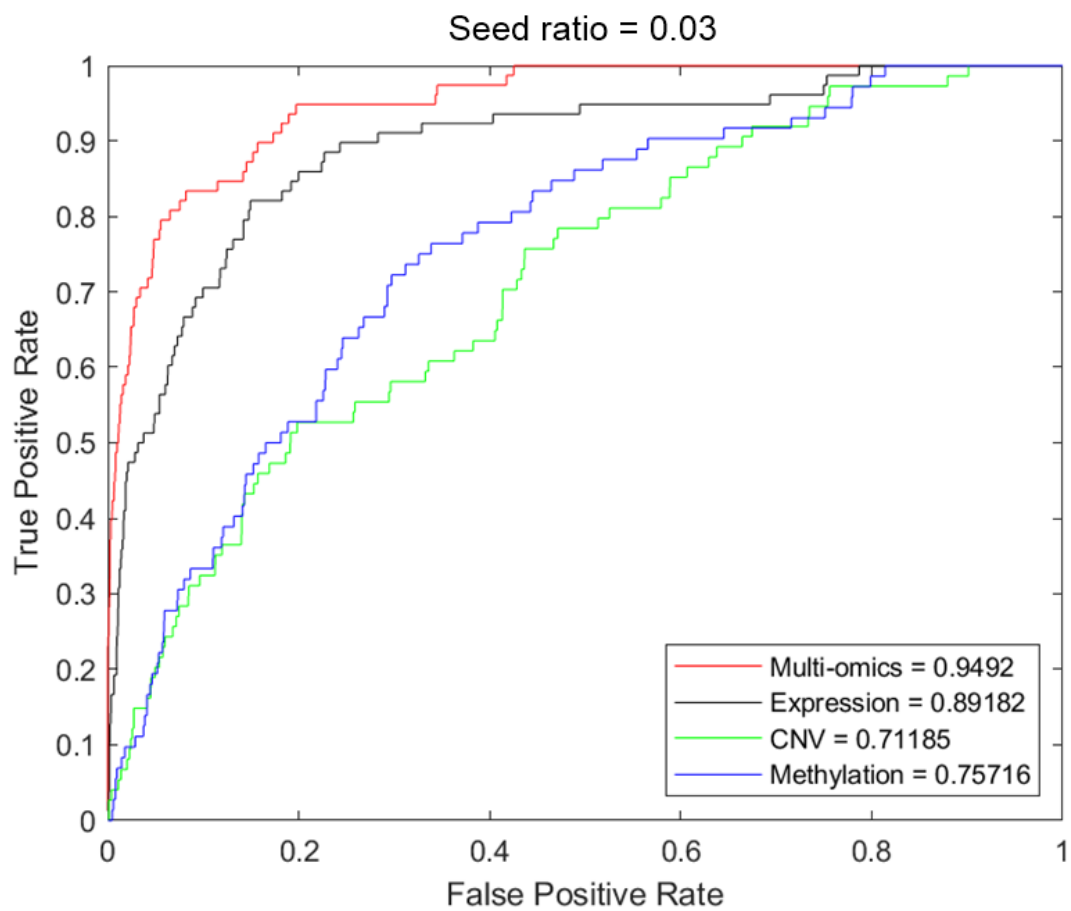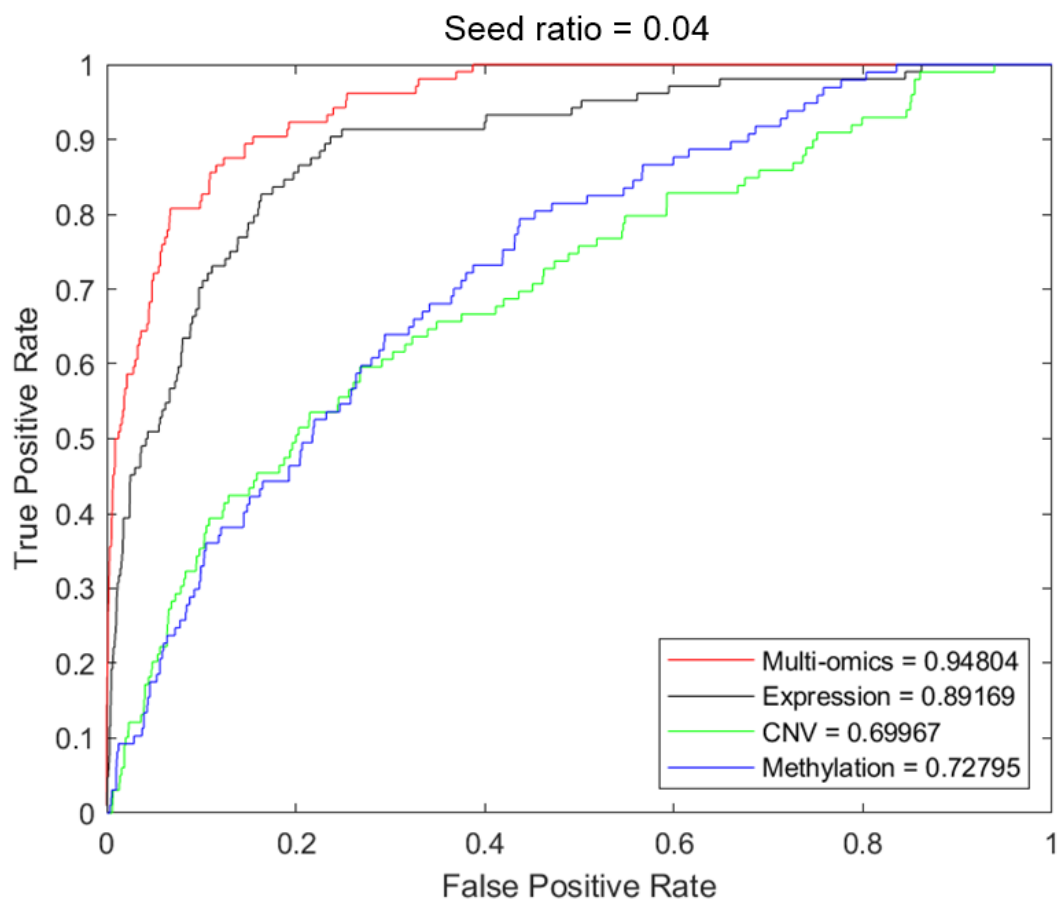

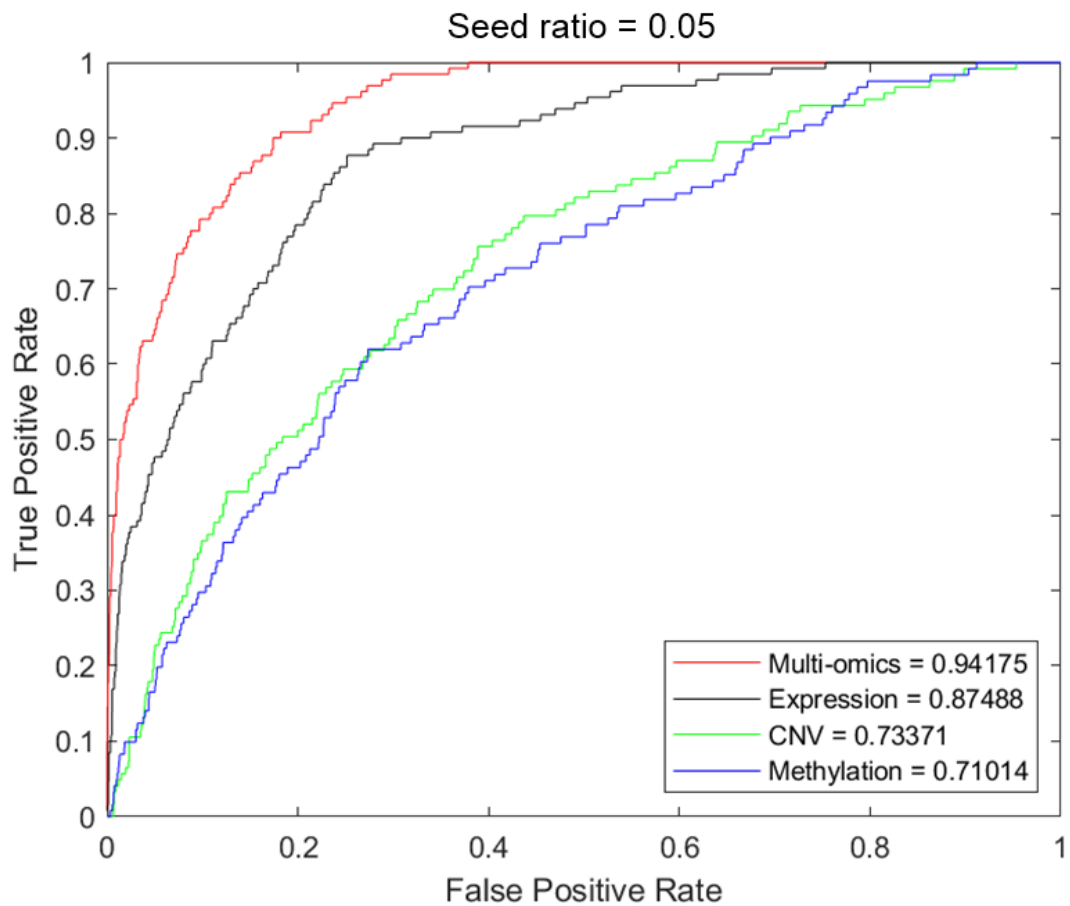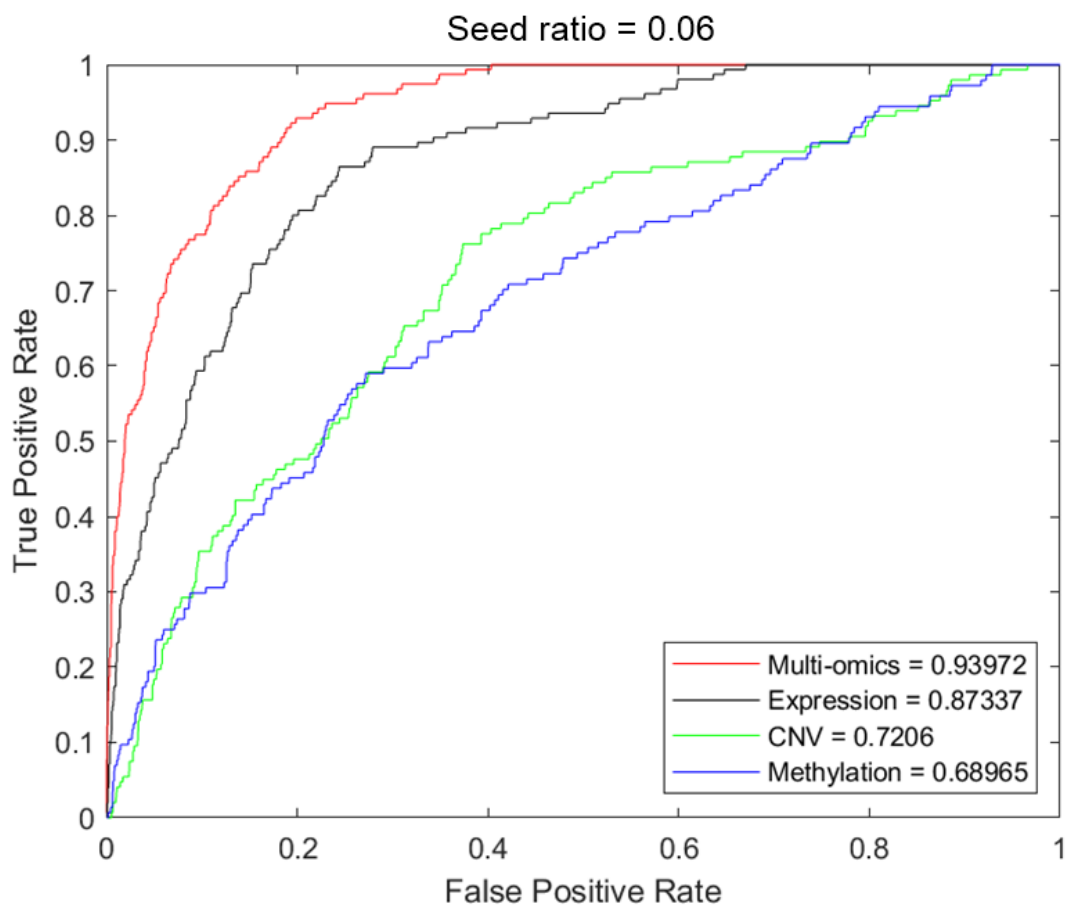

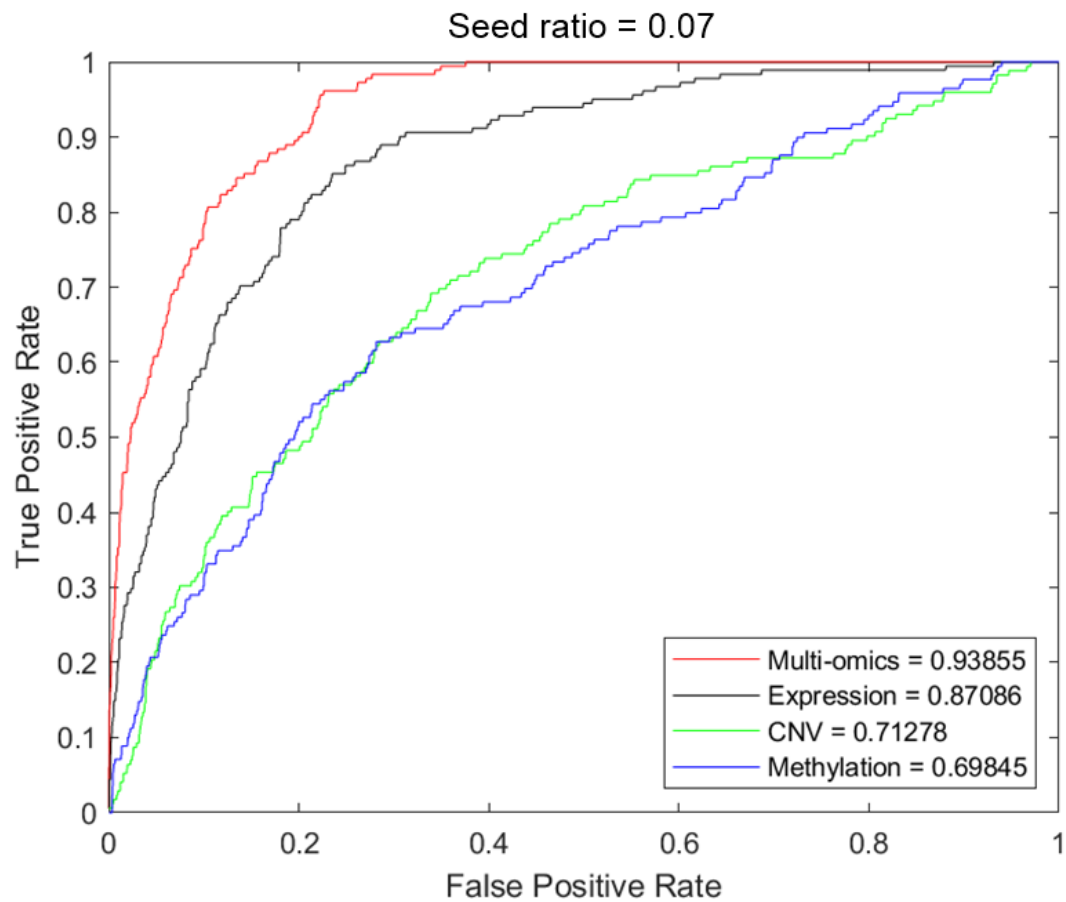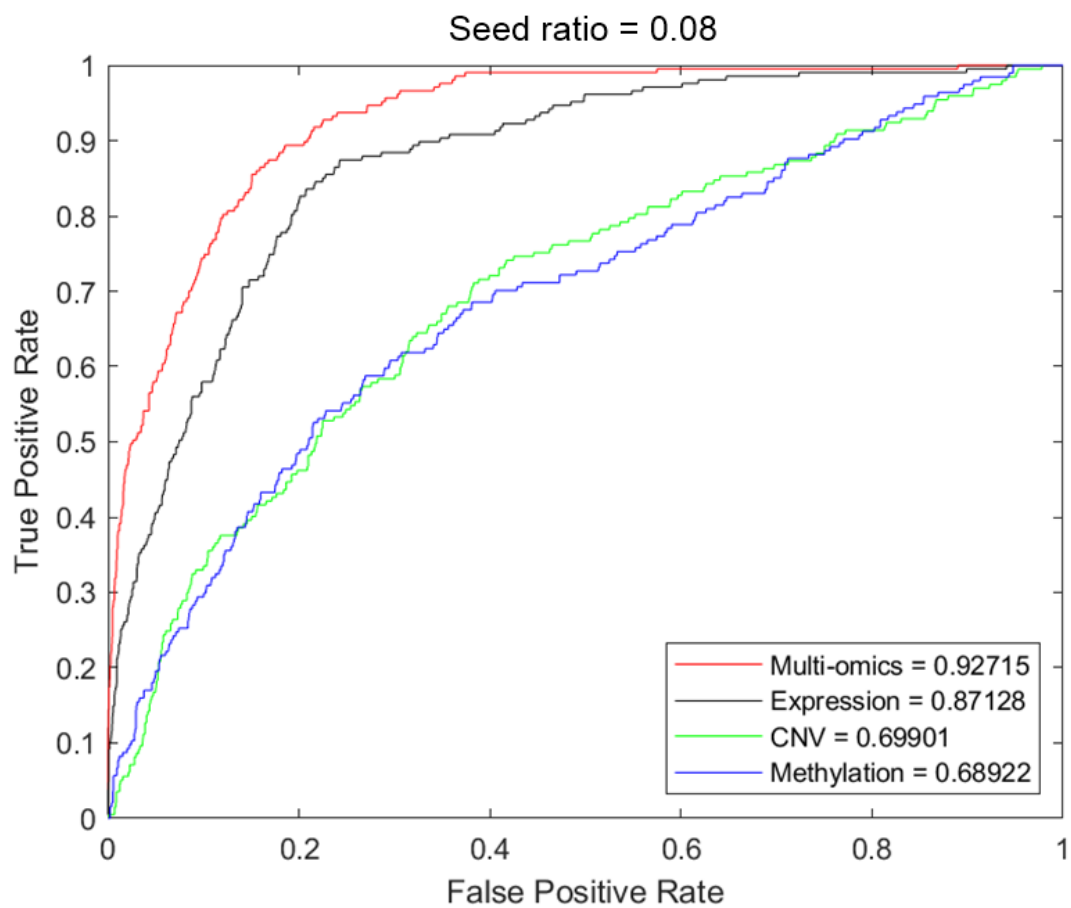

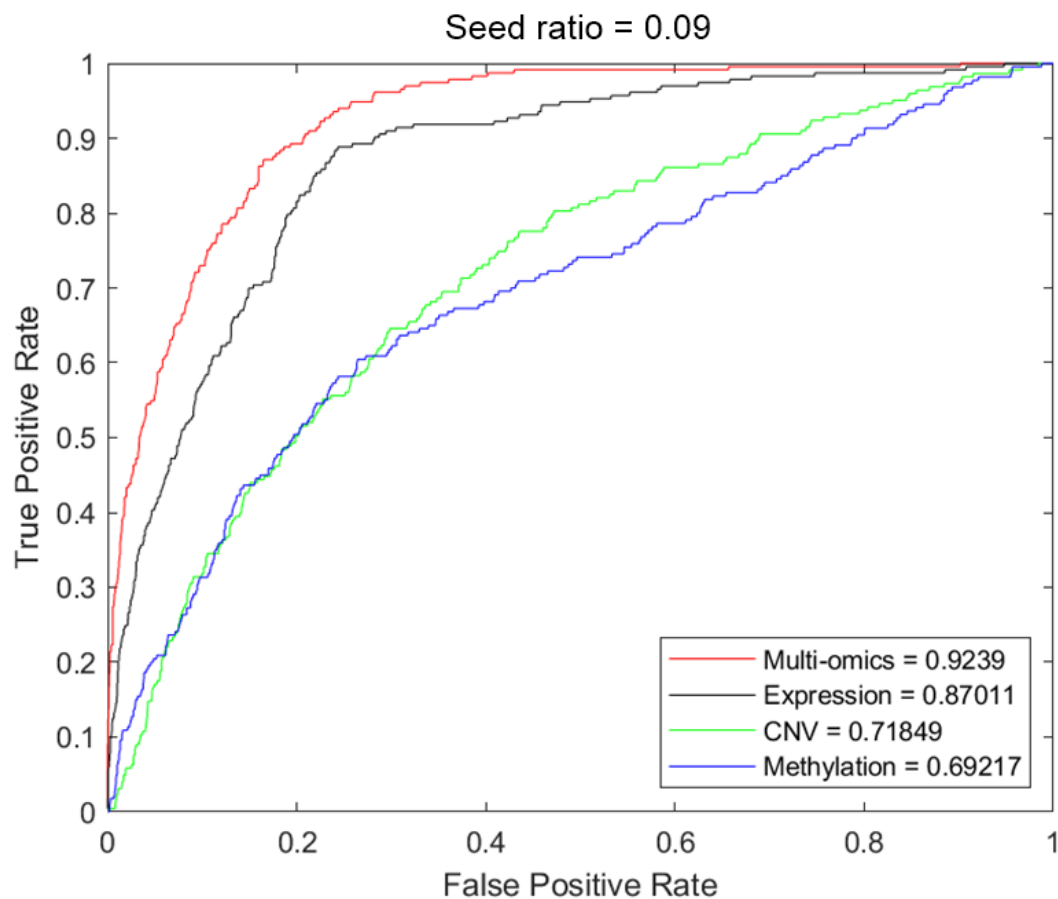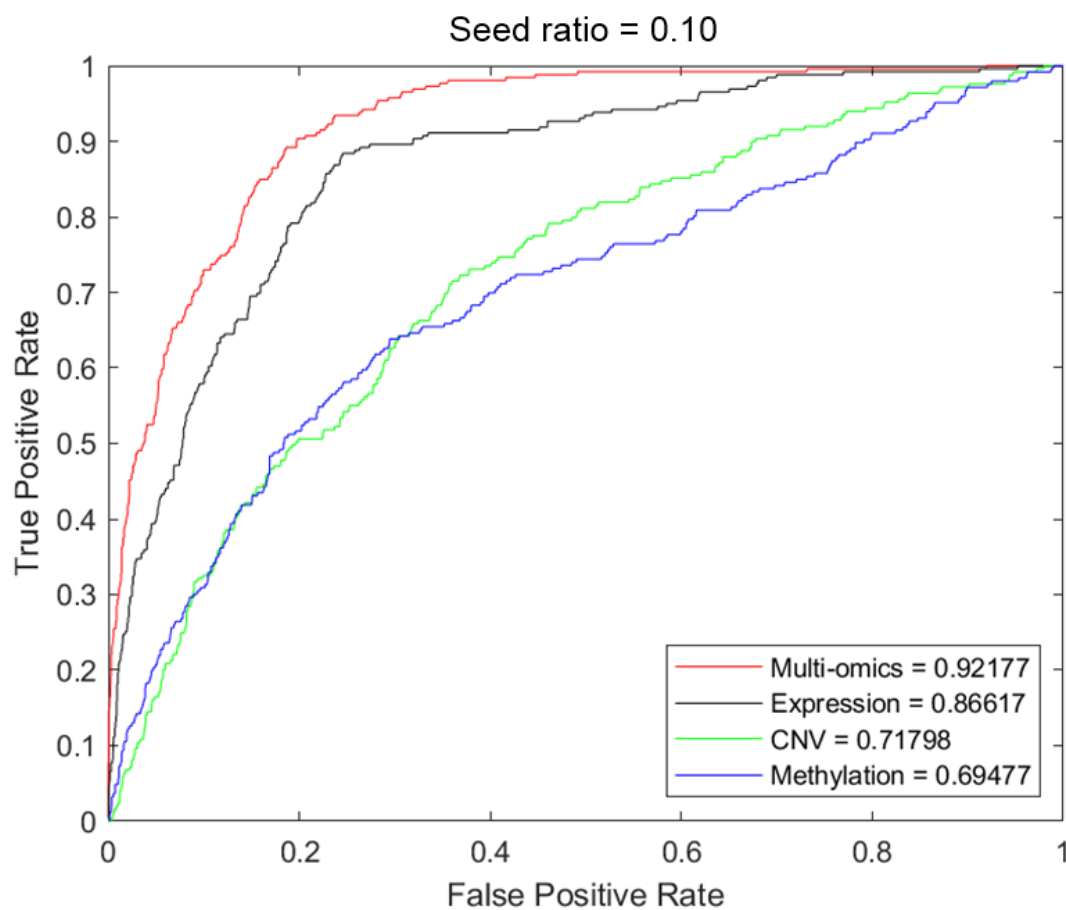

Supplement: Supplementary file 2 — Additional file 2 ROC curve and AUC of the cancer-relevance score of BRCA by various seed ratios. [file 12920_2020_736_MOESM2_ESM.pdf]
