# Supplementary material for: Constructing cancer patient-specific and group-specific gene networks with multi-omics data
Source: BMC Med Genomics. 2020 Aug 27;13(Suppl 6):81. doi: 10.1186/s12920-020-00736-7 (PMC7450550; doi:10.1186/s12920-020-00736-7)
Supplement: Supplementary file 4 — Additional file 4 ROC curve of the cancer-relevance score of each cancer type with the seed ratio of 0.05. [file 12920_2020_736_MOESM4_ESM.pdf]

Additional file 4.

ROC curve and AUC of cancer-relevance score of each cancer type with 0.05 of seed ratio.

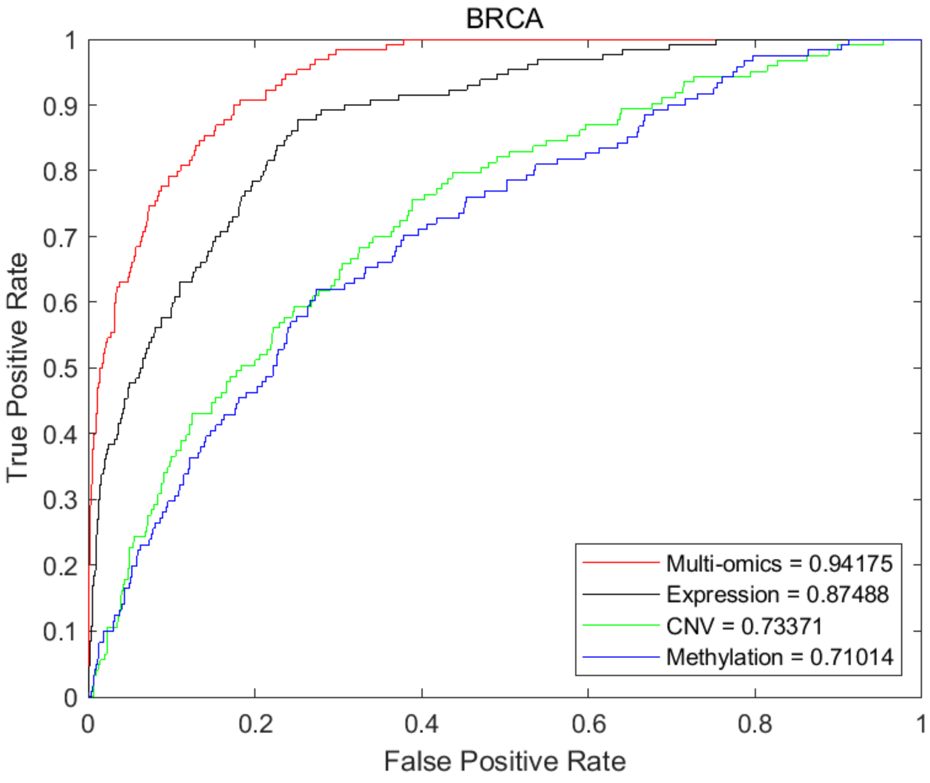

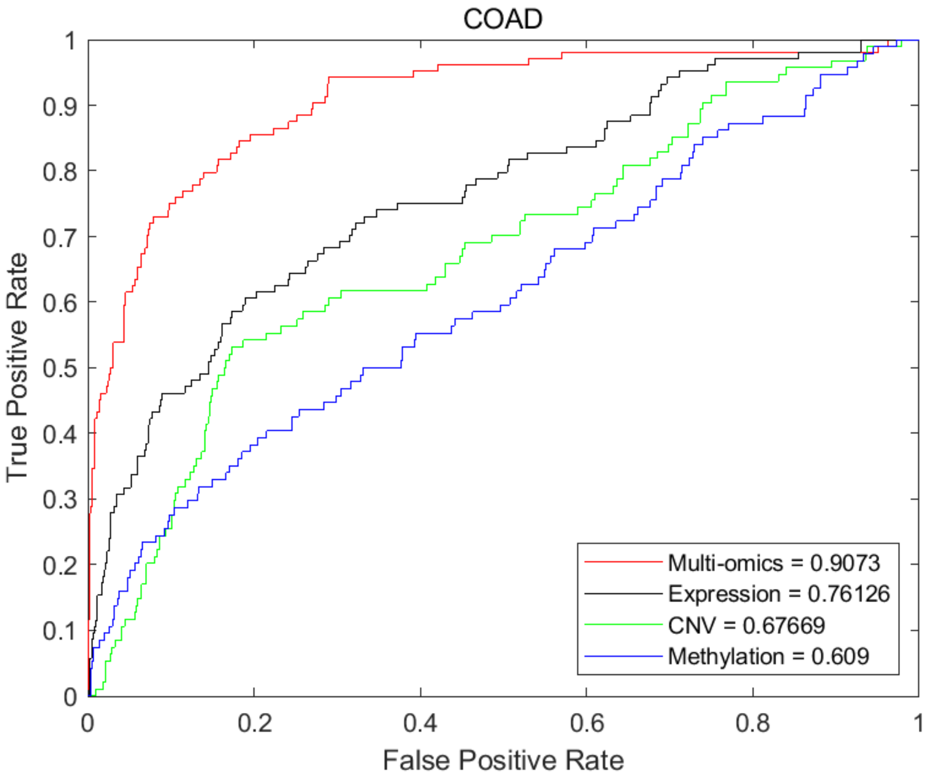

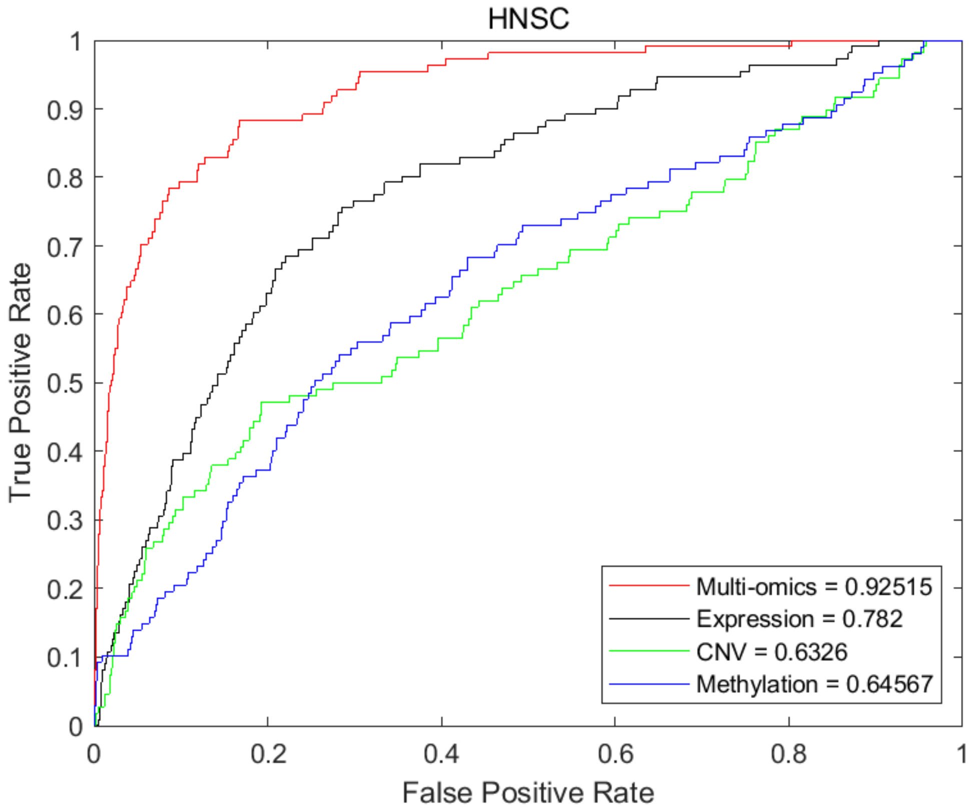
HNSC
True Positive Rate
False Positive Rate
0
0.1
0.2
0.3
0.4
0.5
0.6
0.7
0.8
0.9
1
0
0.2
0.4
0.6
0.8
1
Multi-omics = 0.92515
Expression = 0.782
CNV = 0.6326
Methylation = 0.64567

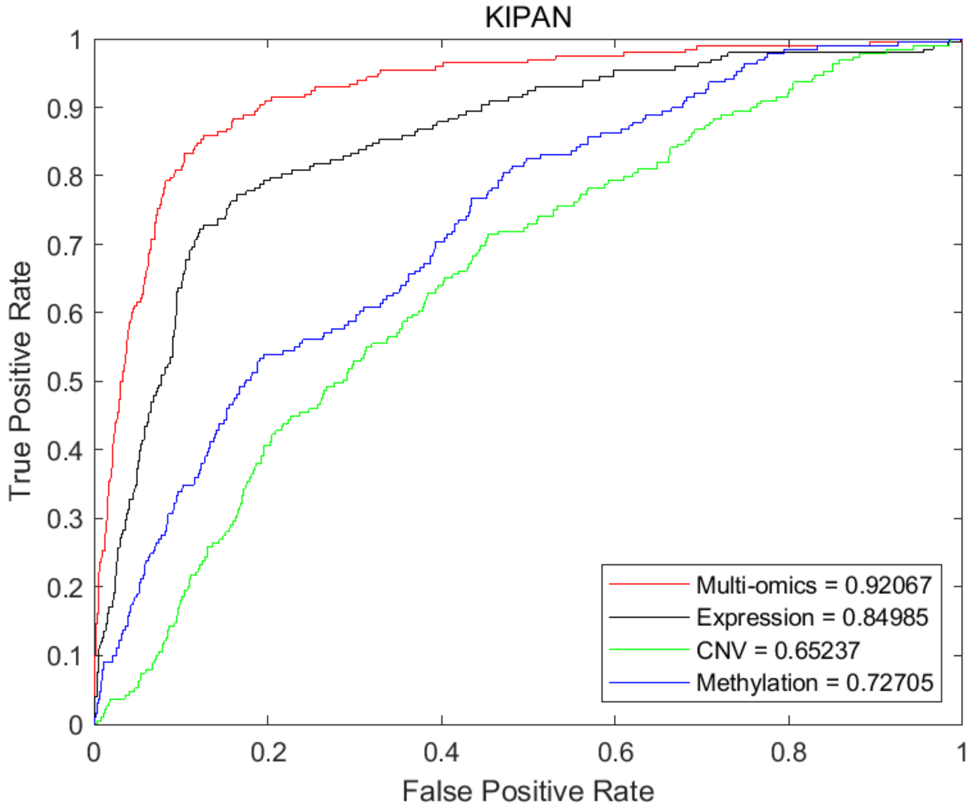
KIPAN
True Positive Rate
False Positive Rate
0
0.1
0.2
0.3
0.4
0.5
0.6
0.7
0.8
0.9
1
0
0.2
0.4
0.6
0.8
1
Multi-omics = 0.92067
Expression = 0.84985
CNV = 0.65237
Methylation = 0.72705

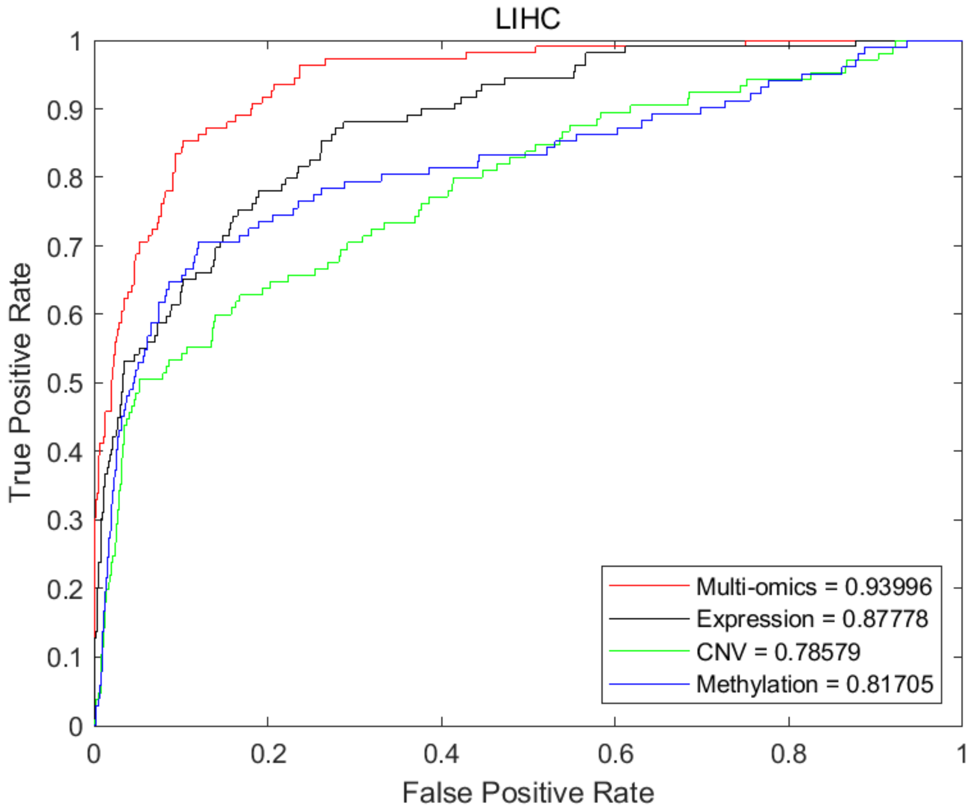

LIHC
True Positive Rate
False Positive Rate
Multi-omics = 0.93996
Expression = 0.87778
CNV = 0.78579
Methylation = 0.81705

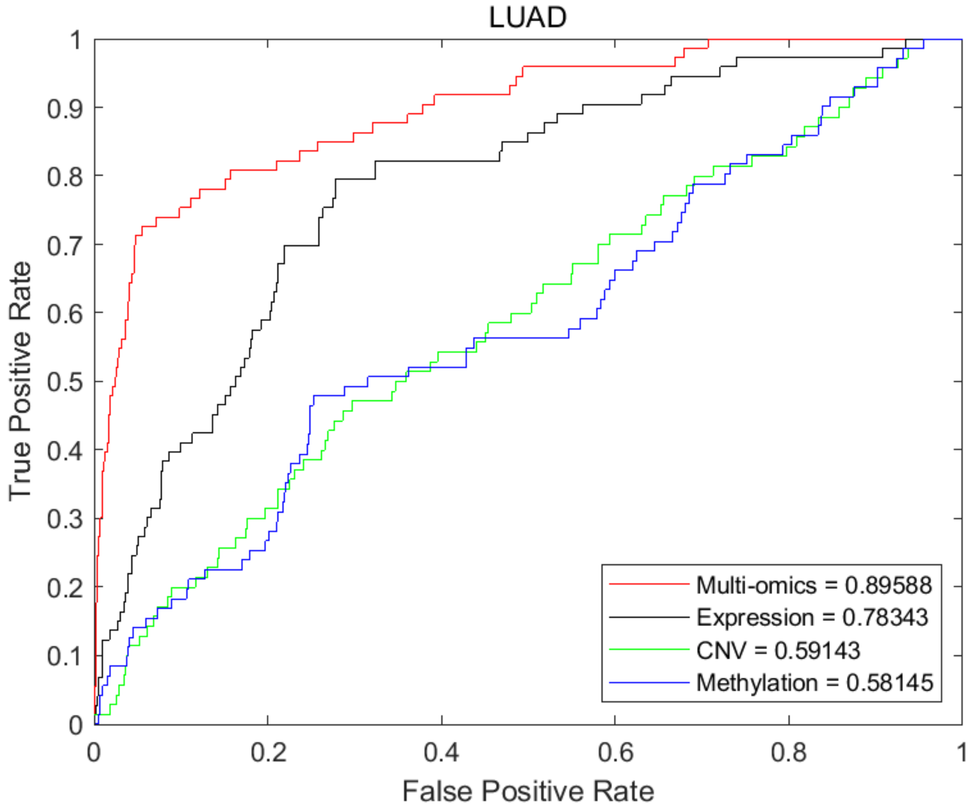

LUAD
True Positive Rate
False Positive Rate
Multi-omics = 0.89588
Expression = 0.78343
CNV = 0.59143
Methylation = 0.58145

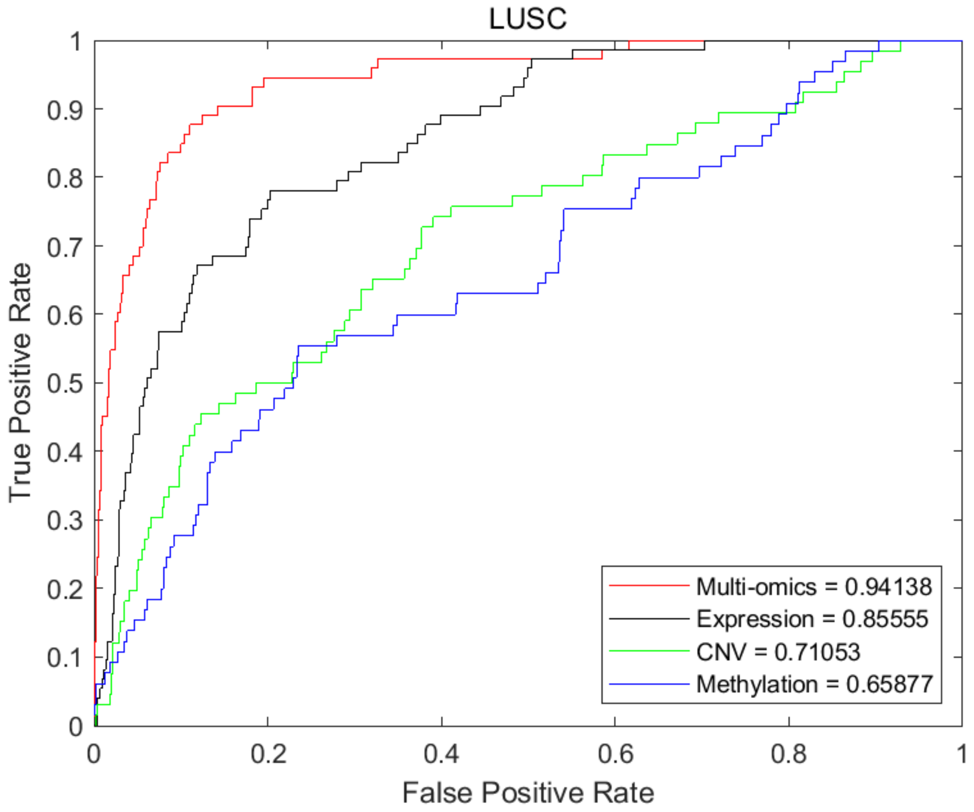
LUSC
True Positive Rate
False Positive Rate
0
0.1
0.2
0.3
0.4
0.5
0.6
0.7
0.8
0.9
1
0
0.2
0.4
0.6
0.8
1
Multi-omics = 0.94138
Expression = 0.85555
CNV = 0.71053
Methylation = 0.65877
